# On Crossover Temperatures of Viscous Flow Related to Structural Rearrangements in Liquids

**DOI:** 10.3390/ma17061261

**Published:** 2024-03-08

**Authors:** Michael I. Ojovan, Dmitri V. Louzguine-Luzgin

**Affiliations:** 1 Department of Materials, Imperial College London, South Kensington Campus, Exhibition Road, London SW7 2AZ, UK; 2 Advanced Institute for Materials Research (WPI-AIMR), Tohoku University, Aoba-Ku, Sendai 980-8577, Japan; dml@wpi-aimr.tohoku.ac.jp; 3 Mathematics for Advanced Materials Open Innovation Laboratory (MathAM-OIL), National Institute of Advanced Industrial Science and Technology (AIST), Sendai 980-8577, Japan

**Keywords:** liquid metals, viscosity, glass transition, crossover temperature, minimal viscosity

## Abstract

An additional crossover of viscous flow in liquids occurs at a temperature *T_vm_* above the known non-Arrhenius to Arrhenius crossover temperature (*T_A_*). *T_vm_* is the temperature when the minimum possible viscosity value *η*_min_ is attained, and the flow becomes non-activated with a further increase in temperature. Explicit equations are proposed for the assessments of both *T_vm_* and *η*_min_, which are shown to provide data that are close to those experimentally measured. Numerical estimations reveal that the new crossover temperature is very high and can barely be achieved in practical uses, although at temperatures close to it, the contribution of the non-activated regime of the flow can be accounted for.

## 1. Introduction

The practical interest in the viscous flow and viscosity of materials at high temperatures is due to many technological applications of molten systems at elevated temperatures, among which the development of liquid metal coolant methods for fast neutron breeder reactors is notable [1,2]. The first metal used in the cooling of liquid metal-cooled fast reactors was mercury (Hg), which was later replaced by the more effective sodium (Na), lead (Pb), a mixture of sodium–potassium (Na-K), and an eutectic mixture of lead–bismuth (Pb-Bi). It is essential that fast reactors enable an effective increase in the energy contained in natural uranium by a factor of 60 to 100, granting the utilization of nuclear power for many thousands of years ahead and ensuring a sustainable nuclear energy supply [1]. The utilization of vitrification is aimed at obtaining bespoke glass and glass crystalline composites for various purposes, and this is another example of how viscous flow affects technology. The cooling of liquids is indeed the main technology intended for producing vitreous materials, with designed compositions including a wide range of oxide glasses (mainly of the silicate family), while the fast cooling of molten metals is currently the basic method aimed toward producing metallic glass [3]. Viscosity reflects the timescale for momentum dissipation and structural relaxation in condensed matter (liquids) and changes by some 16 orders of magnitude in glass-forming systems with respect to relatively small changes in temperature from the liquid state to glass transition temperatures (*T_g_*) [4,5,6,7,8,9,10,11,12]. Attention has primarily been focused on the behaviour of viscosity as a function of temperature in technologies utilizing molten systems, which explains the practical aspects of viscous flow.

Scientific interest in the viscosity and viscous flow of both ordinary matter and systems at extreme conditions is also not diminishing. Unexpectedly, interest in viscosity and the existence of its fundamental lower bound constraint was aroused among quantum gravity, string theory, quark–gluon plasma, and strongly correlated electron system experts [13,14,15,16]. For example, the authors of [17] have shown that there is a certain scale for a lower bound with respect to viscosity, as well as various upper and lower bounds that exist for many other dynamic and thermodynamic properties. 

In the condensed phase of matter, which can be either solid or liquid, the higher the temperature, the higher the concentration of broken bonds; thus, flow is more effortless and viscosity is lower. The viscosities of liquids at high temperatures hence decrease. However, claims that the viscosity at infinite temperature tends toward zero [18] are wrong because, at extremely high temperatures, materials are either in the gaseous or supercritical fluid phase; therefore, their viscosities will not decrease anymore with an increase in temperature but will increase instead [19]. Moreover, since the liquid’s viscosity decreases with increasing temperatures and the gas’s viscosity increases with increasing temperatures, at sufficiently high temperatures, the viscosity must encounter a minimum value [19,20,21,22]. It is notable that viscosity minima were experimentally observed in many non-metallic systems [19,23] and in liquid tin (Sn) [24]. The minimum arises from the crossover between two different viscous flow regimes within condensed and gaseous phases at some crossover temperature, denoted here as *T_vm_*. Namely, this fact has recently allowed Trachenko and Brazhkin to derive a universal equation of the possible minimum viscosities of (non-superfluid) liquids, *η_min_* [20,21]. It has been noted [20] that at very high temperatures, the viscosities of metals are close to 1 mPa·s, and these viscosities are expected to be close to their minima; e.g., this is the case for Fe (2000 K), Zn (1100 K), Bi (1050 K), Hg (573 K), and Pb (1173 K). The quantum mechanical estimations of the possible minimum viscosities of several complex metallic liquids demonstrated that the experimental data are within a difference of one order of magnitude with respect to estimates from the proposed quantum mechanics theory [22]. The minimal viscosities attained in organics were also evaluated in [25], providing results in line with theoretical data. Simultaneously, it was acknowledged in [11,21] that the liquid phase remains poorly understood. The dynamical properties of liquids associated with the non-Arrhenius behaviour of viscosity drastically changed over a relatively narrow temperature range, starting from just above the glass transition temperature (*T_g_*) and ending at a crossover temperature *T_A_,* which is approximately equal although above the liquidus temperature. It is generically accepted that the activation energy of viscous flow *E* is constant below *T_g_* and above *T_A_,* whereas it is a function of temperature *E*(*T*) within the temperature interval *T_g_*–*T_A_*, with many models explaining the non-Arrhenius behaviour of the viscosity within *T_g_*–*T_A_* (see, e.g., [3,4,5,6,7,8,9,10,11,12,26]). The difficulty in such types of treatment and, more generically, describing the thermodynamic properties of liquids is always (see, e.g., [21]) attributed to strong molecular interactions; it is also attributed to the absence of small parameters within theoretical approaches aiming to facilitate calculations and build a thermodynamic temperature dependence as liquids have neither the weak interparticle interactions of gas nor the small atomic displacement characteristics of both crystalline and vitreous solids. One should nevertheless note the significant progress in the development of liquid thermodynamics achieved within the last decade, which is primarily based on the analysis of excitations in liquids [27,28,29,30,31,32]. A recent detailed analysis of viscosity behaviour at high temperatures has, however, shown that the viscosity of liquids is more complex compared with the simplified Arrhenius-type dependence behaviour with a constant activation energy (see, e.g., Figure 1 of [33]). 

The purpose of this paper is to show that the temperature at which viscosity attains its minimal value (*T_vm_*) and minimal viscosity (*η_m_*) can be assessed using extensions of well-tested microscopical viscosity models, such as the Eyring–Kaptay (EK) [34,35] or Douglas–Doremus–Ojovan (DDO) models [7,20,26,36], which present some examples of such calculations. Although our results stand in line with previous works, they present practical interest for the following:

(i) Experimental and practical applications (see, e.g., [1,2]);
(ii) Calculations of the activation energy of flow demonstrating that extremely high viscosity temperature data will not be accounted for via simplified Arrhenius equations used in precise calculations.

## 2. Temperature Crossovers

**Figure 1:**
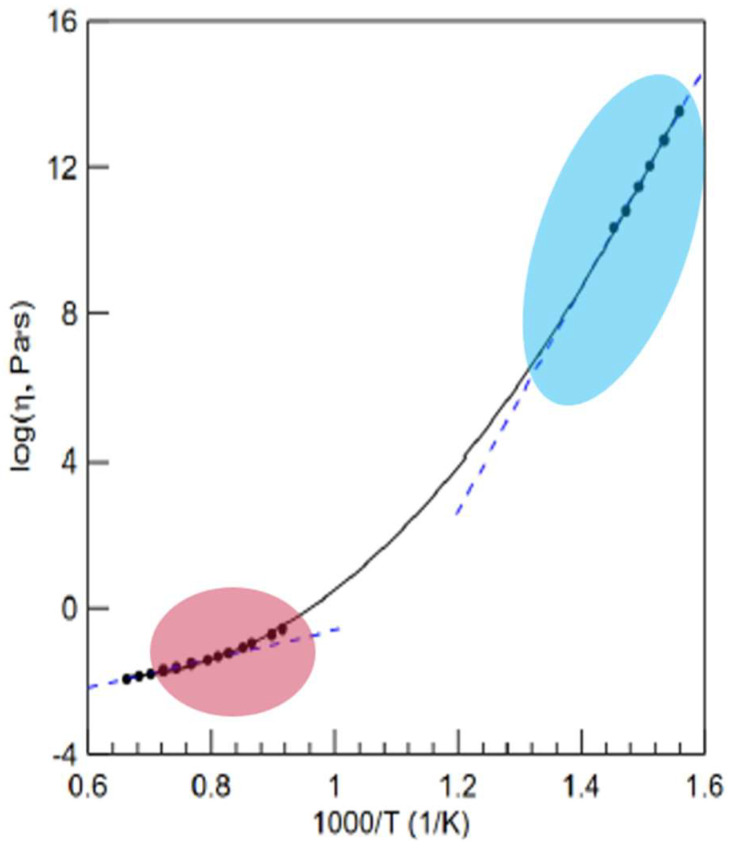
Typical temperature behaviour of the viscosity of amorphous matter shown for the Zr_58.5_Cu_15.6_Ni_12.8_Al_10.3_Nb_2.8_ metallic glass with two distinct Arrhenius-type dependencies at high- and low-temperature ends (modified from [37]). At the low-temperature (high viscosity) end, the metastable liquid turns thermodynamically unstable yet kinetically stable glass, which we consider as a second-order phase transformation. At the high-temperature (low-viscosity) end, the crossover reflects the change from the temperature-dependent structure of a melt to the loose structure of regular liquid.

The typical viscosity temperature behaviour of liquids is illustrated in Figure 1, which shows two distinct Arrhenius behaviour types at high and low temperatures and a temperature-dependent activation energy with respect to flow caused by structural changes occurring in the liquids [37]. Although non-equilibrium viscosity does not follow the slope at low-temperature ranges, as shown in slow enough creep experiments, equilibrium viscosity still follows the Arrhenius law with respect to temperatures and long timescales that are accessible experimentally. 

Following [38], we conclude that the viscous flow in liquids exhibits three temperature ranges:

- Low-temperature (high viscosity) end when *T  <  T_g_*: Arrhenius-type viscosity of glass characterized by the high activation energy of flow *E* = *E_H_*;
- Intermediate temperatures *T_g_ < T < T_A_*: non-Arrhenius-type law formally expressed with an exponent with the variable activation energy of viscosity *E* = *E*(*T*);
- High-temperature (low viscosity) end *T* > * T_A_*: Arrhenius-type viscosity of liquids characterized by a relatively lower (compared to glass) activation energy of flow *E* = *E_L_*.

The first crossover temperature at the high-viscosity end corresponds in practice to glass transition temperature *T_g_*, where the metastable liquid turns into thermodynamically unstable yet kinetically stable glass [36,38]. The first crossover temperature that is set as equal to the glass transition temperature is directly related to the thermodynamic parameters—enthalpy *H_d_* and entropy *S_d_*—of chemical bonds in condensed materials:

(1) $$T_{g}=\frac{H_{d}}{S_{d}+Rln(1-\phi_{c})/\phi_{c}}$$

where ϕ is the percolation threshold that determines when a percolation cluster made of broken chemical bonds—configurons—is formed for the first time [39]. In metallic alloys, due to the non-directional type, these broken bonds can be treated only statistically. 

The second crossover temperature *T_A_* at the low-viscosity end is the temperature above which the liquid becomes fully depolymerized and below which the atomistic dynamics of a liquid become heterogeneous and cooperative; the activation barrier of diffusion dynamics in turn becomes temperature-dependent [36]. The crossover temperature *T*_A_ is in practice assumed to be close to the liquidus temperature *T*_liq_ [38]. A statistical analysis of the existing correlations between *T*_A_, *T*_g_, and the melting temperature (*T*_m_) via artificial intelligence tools showed that regardless of the type of glass-forming liquid, the crossover temperature is given by the following universal equation [40]:

(2) $$T_{A}=kT_{m},$$

where *k* = 1.1 ± 0.15 (see for details Figure 3b of reference [40]). In addition, it is noted that the *T*_A_ of certain glass families, such as float and nuclear waste glass can be defined using a fixed viscosity value that is independent of composition [36,41].

Within the temperature range from *T_g_* to *T_A_*, the activation energy of the temperature dependence of liquid viscosity *E*(*T*) is, in turn, a function of temperature; thus, viscosity exhibits non-Arrhenius behaviour, typically changing its value for the supercooled melts between 10^−2^ and 10^12^ Pa·s (see the vertical axis of Figure 1). It was shown that within this temperature range, configuration entropy (*S*_c_) decreases with decreasing temperatures as glass is increasingly immobilized/vitrified [36]. The most popular equation commonly used to describe the temperature behaviour of viscosity in this range is the Vogel–Fulcher–Tammann (VFT) model, though it completely fails to provide a correct description of viscosity outside the range [3,6,7,9,25,36]. 

**Figure 2:**
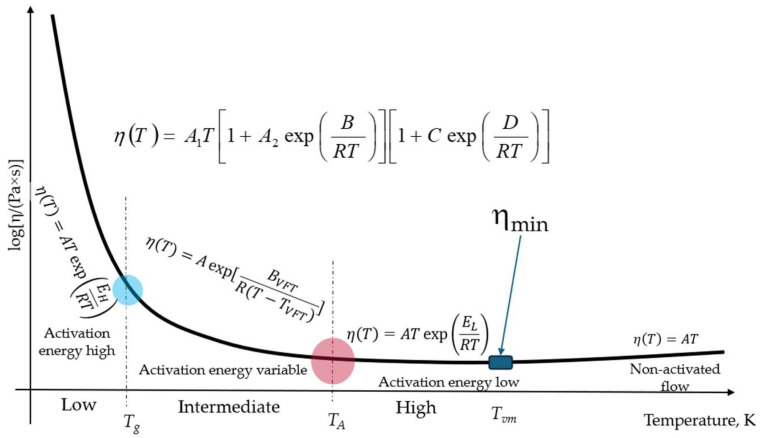
Schematic of the temperature dependence of the viscosity of an amorphous matter at constant pressure (modified after [20]).

The generic behaviour of the viscosity of amorphous materials (glasses and liquids transiting at very high temperatures to either a gaseous or supercritical fluid state) is schematically described in Figure 2.

In line with the latest findings, above *T_A_,* there is a third crossover temperature denoted as *T_vm_* where viscosity reaches its minimum possible value and above which it becomes non-activated, increasing with an increase in temperature. This universally existing crossover should not be confused with polymorphic (liquid–liquid) structural crossovers in metallic systems, such as the temperature-induced changes reported for In, Sn, and Sb [42]. As shown below, the third crossover temperature can be estimated via the following equation:

(3) $$T_{vm}=\frac{H_{m}}{nR},$$

where *H_m_* is the enthalpy of the motion of the configuron, which is identical to the activation energy of viscosity (*E_L_*) (see below), *n* = ½, and *R* is the universal gas constant. 

## 3. Viscosity at the Low-Temperature (High Viscosity and High *E*) End 

The transformation of a liquid when cooling on glass (i.e., vitrification) can take place rapidly enough at melt cooling rates that crystallization is kinetically avoided. Glass transition phenomena are observed universally; moreover, all liquids can be, in practice, vitrified provided that the rate of cooling is high enough to avoid crystallization. The difficulty in understanding the glass transition is because of the absence of obvious changes, i.e., almost undetectable changes in the structure of amorphous materials despite the qualitative changes in characteristics and extremely large changes in the timescale of relaxation processes. The glass transition is experimentally observed as a second-order phase transformation in the Ehrenfest sense with the continuity of the material’s volume and entropy and the discontinuity of their derivatives, which are therefore used in practice to detect where transformation occurs, e.g., to detect the *T_g_* [12,43]. Because of this, the International Union of Pure and Applied Chemistry (IUPAC) defines glass transition as a second-order transition during which a supercooled melt yields, upon cooling, a glassy structure; below the glass transition temperature, the physical properties vary in a manner resembling those of the crystalline phase [44]. Experimentally, the glass transition is observed as a second-order-like phase transformation, and discontinuities are observed only for their derivatives. Due to the universally observed thermodynamic evidence of second-order-like phase transformations in amorphous materials upon a change in temperature at the glass transition, the term “calorimetric glass transition” was coined—see Chapter 3.2 of Ref. [43]. The crucial argument for treating vitrification as a phase transformation of amorphous materials at *T_g_* is related to the possibility of observing structural changes at the glass transition. Obvious symmetry changes occur at crystallization, with the formation of an ordered (most often periodic, although for quasicrystals not necessarily) anisotropic structure. The structure of glass is, however, disordered, resembling that of liquids (though somewhat more ordered at the medium range scale of 0.5–1 nm [45]). It is difficult to structurally distinguish glass from a melt near *T_g_* based on the distribution of atoms and using available techniques, such as X-rays or neutron diffraction. A breakthrough in understanding structural differences between glasses and liquids at temperatures below and above *T_g_* constituted the work of Kantor and Webman [46], who have proved that the rigidity threshold of an elastic percolating network is identical to the percolation threshold. Analysing the structure of chemical bonds between atoms that constitute condensed matter and focusing on the behaviour of broken bonds termed configurons in a condensed matter [47,48], one can identify the percolation threshold as a function of temperature and thus find the critical temperature when solid-like behaviour changes to liquid-like behaviour [20,39]. For amorphous materials, *T*_g_ is thus assigned to the temperature when percolation via configurons occurs upon heating. Based on the Kantor–Webman theorem (i.e., using the conclusion that the rigidity threshold of elastic networks is identical to the percolation threshold [46]), the configuron percolation theory (CPT) treats the transformation of glass into liquids at *T*_g_ as an effect resulting from percolation via configurons and provides explicit evidence on the different structural arrangements of glass compared to liquids [49]. The CPT envisages that the structural arrangements of melts (highly viscous supercooled liquids above *T_g_*) and glasses (*T < T_g_*) are different. The physical picture of the glass transition in amorphous materials involves the representation of a topological change in a disordered bond lattice network. Melts exhibit a fractal geometry of configurons, with broken bonds forming extended percolation (macroscopic) fractals, and because of this, they exhibit liquid-like behaviour. In contrast, just below *T_g_,* glass exhibit a 3D geometry of bonds, with point-type broken bonds having a nil-dimensional (*D* = 0) geometry, and because of this, they exhibit solid-like behaviour. Namely, set theory, which is a branch of mathematical logic that studies abstract sets, properly characterizes structural changes at the glass transition, providing unambiguous proof that the set of configurons behaves differently in glass and melts: The set of configurons changes its Hausdorff–Besicovitch dimensionality at the glass transition temperature from 0 in the glass to *D* = 2.55 ± 0.05 (fractal) [see Table 3 in Chapter 3.1 of Ref. [3]]. 

The CPT also concludes the physical absence of the Kauzmann entropy catastrophe associated with its temperature, which exactly conforms with the conclusions of Mauro et al., who doubts its existence as well [6]. 

Data from neutron or X-ray diffractometry explicitly reveal the almost undetectable changes in the structure of amorphous materials at the glass transition [50,51,52]. The most effective is the analysis of the temperature behaviour of the first sharp diffraction minimum (FSDM) rather than the first maximum value of pair distribution functions (PDFs) [51,52]. Both the amplitude and position of FDSM (*PDF_min_*) depend on the temperature being shifted with different rates above and below *T_g_* (see Figure 1 of [42] and especially Figure 1 of Ref [51]). The temperature changes in FDSM are linear with respect to temperature, exhibiting just a kink at *T_g_,* while the rate of growth *d*(*PDF_min_*)/*dt* changes stepwise from a lower value to a higher one at *T*_g_, exactly the same as the Hausdorff–Besicovitch dimensionality of configuron set (see Figure 4 of Ref. [52]). The temperature behaviour of materials near *T_g_* is hence explained based on the concept that supercooled liquids (below *T*_l_) continuously change their atomic arrangements upon cooling [37]. 

Formally, the CPT is a two-state model in which the high energy level is the configuron phase and the low one is represented by the intact bonds operating on a bond lattice instead of the more conventional particle lattice; because of this, the coupled thermodynamic modelling of the glass transition is possible, enabling a formal description of the main features of the glass transition, including the hysteresis loop of the heat capacity detected by DSC in the glass transition range during cooling/reheating cycles at various rates [53]. The universal equation of viscosity (the double exponential DDO model) of both solid and liquid amorphous materials provided by CPT is valid over the whole temperature range (see Figure 2) [7,20,25,36]:

(4) $$\eta (T)=ATexp(\frac{H_{m}}{RT})[1+Cexp\left(\frac{H_{d}}{RT}\right)]$$

where *A* = *A*_1_*A*_2_, *A*_1_ = *k_B_*/*6πrD*_0_; *k_B_* is the Boltzmann constant; r is the configuron radius; *A*_2_ = exp(−*S_m_*/*R*), *C* = exp(−*S_d_*/*R*), and *D*_0_ = *fg**λ**^2^zp*_0_*ν*_0_; *H_d_* and *S_d_* are the enthalpy and entropy of the configuron formation; *H_m_* and *S_m_* are the enthalpy and entropy of the configuron motion (see Table 3 of Ref. [20] for numerical data of a range of materials); *f* is the correlation factor; *g* is a geometrical factor (~1/6); λ is the average jump length; z is the number of nearest neighbours; *p*_0_ is a configuration factor; and *ν*_0_ is the configuron’s vibrational frequency. Equation (4) gives a correct description of viscosity with two exact Arrhenius-type asymptotes below and above the glass transition temperature, whereas near *T*_g_, it practically gives the same results as well-known and widely used viscosity equations, e.g., the VFT model. Deviations in the temperature’s variable activation energy from smooth functions or asymptotic Arrhenius constants noted in [33] could potentially be connected with polymorphic structural rearrangements and crossovers, as reported in [42]. At *T < T_g_*, Equation (4) simplifies into an Arrhenius-type law *η*(*T*) = *A·C·T·exp*[(*H_m_* + *H_d_*)/*RT*], indicating that at the low-temperature (high viscosity) end, the activation energy is constant and high: *E_H_* = *H_d_ + H_m_*. 

## 4. Viscosity at the High-Temperature (Low Viscosity and Low *E*) End 

The CPT approach can also be used to extend the analysis of structural changes in liquids to the high-temperature range, where the low-viscosity crossover reflects a change from the temperature-dependent structure of a melt to the loose structure of a regular liquid [36,54,55,56]. At *T* > *T_A_*, Equation (4) simplifies to *η*(*T*) = *A·T·exp*(*H_m_*/*RT*), indicating that at the high-temperature (low viscosity) end, the activation energy of the flow is constant and low: *E_L_* = *H_m_*. Observing that *ν*_0_ = *(k*/*m)*^1/2^/2π, where *k* is the force constant of the oscillating configuron near its equilibrium position [57], *m* is its mass, λ = (6 *v*/π)^1/3^, *r* = (3 *v*/4*π*)^1/3^, *v* is its volume, and using notation *A* = *πk_B_exp*(*−S_d_*/*R*)/*fgzp*_0_*k^1^*^/*2*^, one can rewrite the DDO equation at *T* > *T_A_* as follows:

(5) $$\eta (T)=A\frac{M^{1/2}{\;T}^{n}}{V^{m}}exp(\frac{E_{a}}{RT}),$$

where *n* = 1 and *m* = 1 and the activation energy of viscous flow within CPT is *E_a_* = *E_L_* = *H_m_*. This form of the DDO equation describes the well-known Eyring equation (see Equation (22) in [34]) separately from the pre-exponential temperature dependence, which is the square root of the temperature rather than exhibiting linearity for the Eyring equation. Moreover, expression (5) coincides with Frenkel’s equation of the viscosity of liquids [58], see, e.g., Equation (7.14) and the derivation in [59], which exhibits linear behaviour with respect to the temperature pre-exponent. It was correctly noted in [33] that the transition rate applied in the Eyring analysis is more precisely expressed via the linear dependence on the temperature of the pre-exponent (see Equation (2) of [33]) as provided by (5) rather than *T*^1/2^. Nevertheless, the Kaptay equation [35], which is widely accepted and tested on many liquid metals, is very similar to the original Eyring equation concerning the square root temperature dependence:

(6) $$\eta (T)=A\frac{M^{1/2}{\;T}^{1/2}}{V^{2/3}}exp(\frac{E_{a}}{RT}),$$

We can therefore retain form (5) for our analysis, accounting for the DDO model [20] we have *n* = 1, and for the Eyring–Kaptay (EK) [35] model one shall use *n* = 1/2. The pre-exponential term, which is set as power-dependent on temperature (*T^n^*, where *n* = 1 or *n* = ½), is present in the viscosity equations in many models—see, e.g., references [3,4,5,6,7,8,9,10,20,25,35,36,38,39,59,60]—although the experiments are not accurate enough to conclude on exact value of power term (*n*) and distinguish whether this factor is really needed (see, e.g., Doremus’s comments about silica glass in Chapter IV of [59]).

Both Equations (5) and (6) show the presence of the minimum viscosity given generically by

(7) $$\eta_{min}=\eta (T){\left(\frac{T_{vm}}{T}\right)}^{n}exp(n-\frac{nT_{vm}}{T}),$$

which is reached at the temperature given by the following equation:

(8) $$T_{vm}=\frac{E_{a}}{nR}$$

Equation (7) enables an assessment of *η*_min_ from any known viscosity *η(T)* at *T* > *T_A_*, whereas Equation (8) shows a simple relationship between the activation energy at *T* > *T_A_* and *T_vm_*: the higher *E_a_*, the higher *T_vm_*. The difference between DDO and EK models is observed with respect to the pre-exponent temperature-dependent term, where for the first term, *n* = 1 and for the last term, *n* = 1/2. The exact power dependence in the pre-exponent term in (5) cannot be set a priori in our analysis because both EK and DDO models are used here in the extended range of temperatures when they can become inexact. Thus, the power term *n* in Equation (5) has to be identified through an analysis of experiments, the number of which is currently not enough for conclusions. A comparison of the temperatures of viscosity minima taken from NIST [23] with those estimated using (8) shows that the experiments are better described, assuming that *n* = 1/2 (see data below for metals that also conform better with Equations (7) and (8) at *n* = 1/2). 

## 5. Results

**Figure 3:**
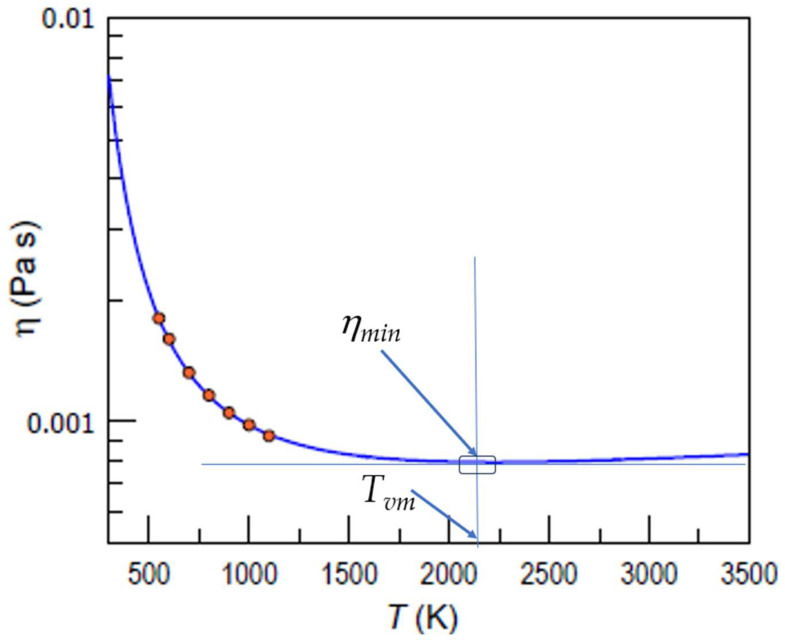
The temperature dependence of the viscosity of Sn calculated via the EK model (blue curve), based on experimental data (orange circles) and adapted from [37] to explicitly show *η_min_* and *T_vm_*.

The viscosity minima, well known for many substances available in the NIST Chemistry WebBook [23] (see also Figure 1 of Ref. [19]), are not yet confirmed experimentally for metallic systems, and they are only predicted by calculations in available publications such as [19,21,22,33,35,37]. The available data on the viscosity of liquid Sn at high temperatures, such as [61,62,63], did not reveal the viscosity minimum, only enabling a rough appreciation of the asymptote of viscosity by typically taking the highest-temperature experimental data for metals as an approximate measure of the minimum viscosity [22]. Figure 3 shows the predicted minimal viscosity of liquid Sn based on the available data at the publication time of [37].

**Figure 4:**
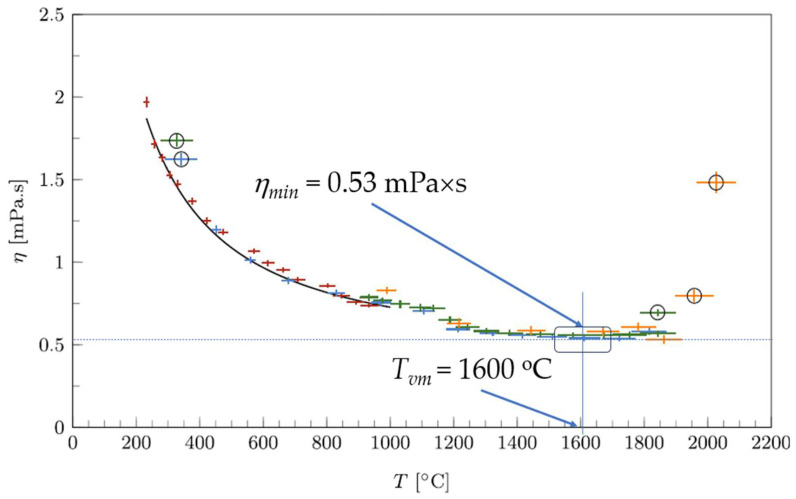
The viscosity of liquid tin as a function of temperature. Although the circled values are considered as failed data, the minimum viscosity is clearly observed and an assessment of both minimum viscosity and the temperature at which this was achieved is possible; this was adapted from [24] to explicitly show *η_min_* and *T_vm_*. Reproduced with modifications from [24] with the permission of Elsevier.

The viscosity of several molten metals was recently measured over a wide temperature range up to 2100 K using an oscillating cup viscometer [24]. The viscosities of both Sn and Pb were successfully measured over the temperature range 506–2135 K for liquid tin and 710–1770 K for liquid lead, providing unpublished data and explicitly revealing the presence of the minimal viscosity of Sn at about 1600 °C (Figure 4). 

Calculations were carried out based on data for the activation energy *E_L_* of viscous flow. Here, *E_a_* = 7.15 kJ/mol [24], and *T_vm_* = 1720 K and *η_min_* = 1.1 mPa·s are given, both being quite close to the experimental results of [24] at 1870 K and 0.53 mPa·s. Moreover, the activation energy of the flow is most probably underestimated because of the calculation of data close to *T_vm_* where the slope of the viscosity curve is smaller because of the pre-exponent term rather than the exponential term in (7). A higher *E_a_* would result in both *T_vm_* and *η_min_* values being closer to the experiment. Anyhow, these results confirm that the pre-exponential parameter of temperature dependence is better described by square root dependence on temperature rather than linear dependence, which would result in twice lower temperatures. Table 1 shows the calculation results of the expected minimal viscosities of some liquid metals of interest. 

The absolute value of viscosity, including its minimum, depends on multi-body interactions between the molecules (or atoms) of liquids, which differ significantly from the case of gas; therefore, it is difficult to envisage the trends of its behaviour. 

## 6. Discussion

Equation (8), which interlinks *T_vm_* with *E_a_*, indicates that for many metallic glasses, such as those discussed in detail in [22], the expected *T_vm_* values are extremely high for any practical application, and even experimental measurements are questionable, which is demonstrated by Zr_57_Nb_5_Cu_15.4_Ni_12.6_Al_10_ in Table 1. An additional difficulty in the detection of minimum viscosity and the corresponding *T_vm_* is the shallow character of the minimum, which makes the identification of actual data rather inexact. It should be noted that viscosity minima are perceived as obvious only in the log–log scale for temperatures, such as those shown in Figure 1 of [20], whereas on a linear scale (see Figure 2, Figure 3 and Figure 4 above), the minima are located within a long interval of temperatures where viscosity is almost unchangeable. Namely, this shallow range of almost unchangeable viscosity serves as an incentive to suppose that there is asymptotic viscosity attained at *T* → ∞ [6,10,12,38], whereas in reality, as it was earlier stated in [20] and proved by Trachenko and Brazhkin [19], viscosity has a minimum value, after which it increases with an increase in temperature, although this increase is quite gradual near *T_vm_*. 

The processing of experimental data on the viscosities of melts at very high temperatures accounts for the fact that the viscosity curve exhibits a change in its slope upon approaching *T_vm_* not only because of a decrease in the exponential term in (5) but because of a significant increase in the pre-exponent of (5). Due to this, in the typical case of calculations when the flow is approximated by a simple Arrhenius curve, *η*(*T*) = *η*_0_*exp*(*Ea*/*RT*), with a temperature-independent pre-exponent η_0_, the activation energy of the flow obtained, *E_a_*, will be always lower in experiments, which accounts for the data closer to *T_vm_* compared to those that are far from it. Table 2 shows the melting and crossover temperatures, including the newly introduced temperature above which the atomic flow becomes non-activated. 

Correlations between various parameters of materials, including crossover and melting temperatures, are effectively revealed when applying artificial intelligence tools [40,72,73], while this is questionable when using unknown data on the actual *T_vm_*, as observed in the last column of Table 2. This is nevertheless essential, and an important aspect in finding correlations between, e.g., *T_vm_* and *T_m_* would be establishing the best value of parameter *n* in Equations (5) and (8), which we took as 0.5 in our assessments despite serious arguments for using *n*= 1 instead (see, e.g., Tables II and III of [60]). The ratio of *T_vm_* to *T_m_* in Table 2 shows the departure of crossover temperatures from the melting point and thus allows a tentative appreciation of the experimental possibility of attaining *T_vm_* in an envisaged experiment. From the data shown in Table 2, it may be observed that the minimum viscosity can occur where the measurement is impracticable, e.g., for Pb and, moreover, for Zr_57_Nb_5_Cu_15.4_Ni_12.6_Al_10_ when *T_vm_* values are extremely high. However, a comparison of crossover temperatures and the temperatures when metallic systems start boiling, *T_b_*, shows that the experimental identification of *Tvm* is possible for others, e.g., *T_vm_*/*T_b_* = 0.84, 0.6, and 0.34 for Bi, Sn, and Ga, respectively. Moreover, this would be possible if Equation (5) is at *n* = 1, similarly to the original DDO model, and the predicted *T_vm_* values are 50% lower, thus falling within the reach of common viscometers.

Another crucial aspect of metallic systems operated in fast neutron reactors is the effect of radiation [1,2]. It is experimentally known that radiation affects the viscous flow; moreover, it significantly changes the character of viscosity, lowering the activation energy of the flow in condensed matter from the high value typical of glass to a low value, which is characteristic at high temperatures: *E_H_* → *E_L_* [74,75,76]. This results from the fact that irradiation breaks down interatomic bonds, thus facilitating the flow similarly to how the temperature does. Therefore, it is highly unlikely that irradiation changes the viscosity at high temperatures when most interatomic bonds are already broken due to the effect of high temperatures. 

The structural changes that occur in liquids at temperatures above the liquidus temperature are behind the experimentally observed changes [42,54,55,56,70,71]. However, upon a further increase in temperature, the matter is at a state between the true condensed phase and the gaseous phase if it is not in the supercritical phase, which is when there is no distinction between them. In many cases, the structure of matter at temperatures approaching and exceeding *T_vm_* is similar to no man’s land, with very little experimental data available to conclude which models are most appropriate for use in calculating viscosities. The equations used to assess the flow at these temperatures are extensions of known models, which are proven to work for condensed matter, and because of this, there is a potential chance that they will always properly provide reliable and more or less exact data for describing the experiment. New insights are needed in this range, and new approaches can be used to analyse the disordered matter, such as the utilization of persistent homology as a type of topological data analysis [77,78,79].

## 7. Conclusions

Liquids exhibit at least three temperature intervals: Viscosity changes its character from an Arrhenius regime at low temperatures to a non-Arrhenius regime at intermediate temperatures and then back to the Arrhenius regime at high temperatures; however, this occurs with a lower flow activation energy (see Figure 1 and Figure 2). Moreover, the higher the change in activation energy, the more fragile the liquid. There is, however, an additional crossover temperature at extremely high temperatures when viscosity attains its minimum possible value; here, the flow becomes non-activated and starts to increase with temperature. The equations proposed for assessing the minimum viscosity (7) and the new crossover temperature (8) are shown to provide reasonable data that are close to those observed experimentally in [23,24].

## Figures and Tables

**Table 1:** Expected minimal viscosities and temperatures at which this is expected to occur ^1^.

Metal	*T* ^2^, K	*η(T)*, mPa·s	*E_a_*, kJ/mol	*T_vm_*, K	*η_min_*, mPa·s
Hg	273	1.55	2.51	604	1.26
Na	723	0.25	5.24	1260	0.23
K	337	0.51	5.02	1210	0.26
Pb	600	2.04	10.43	2510	0.85
Bi	723	1.28	6.45	1550	1.06
Ga	1000	0.62	3.8	920	0.62
Sn	505	1.97	7.15	1720	1.1
Zr_57_Nb_5_Cu_15.4_Ni_12.6_Al_10_	1538	11	65.8	15,840	0.37

^1^ Data on activation energies and viscosities at the indicated temperatures were taken from [24,64] for Sn and Pb; from [1,64] for Na, Pb, and Bi; from [64,65] for Hg; from [66] for Ga; and from [22] for Zr_57_Nb_5_Cu_15.4_Ni_12.6_Al_10_, with *E_a_* assessed from Figure 1 of [22]. ^2^ The temperature T is the temperature at which viscosity (in column 3) is used for calculations in Equation (7).

**Table 2:** Melting (*T_m_*) and crossover temperatures *T_g_*, *T_A_*, and *T_vm_* at which the character of viscous flow changes ^1^. Below *T_g_*, viscosity is characterized by a high temperature-independent activation energy *E_H_*. Between *T_g_* and *T_A_*, the activation energy of the flow is a function of temperature *E*(*T*), decreasing with its increase. Above *T_A_*, the activation energy is temperature-independent, and a low *E_L_* is observed. At *T_vm_*, viscosity attains the minimum possible value, and the flow becomes non-activated with an increase in viscosity upon an increase in temperature.

Metal	*T_m_*, K	*T_g_*, K	*T_A_*, K	*T_vm_*, K	*T_vm_*/*T_m_*
Hg	234	169	257	604	2.6
Na	370	189	407	1260	3.4
K	337	166	371	1210	3.6
Pb	600	..?..	660	2510	4.2
Bi	544	202	598	1550	2.8
Ga	303	97	333	920	3
Sn	505	159	556	1720	3.4
Zr_57_Nb_5_Cu_15.4_Ni_12.6_Al_10_	1123	671	1349	15,840	14.1

^1^ Melting temperatures are taken from [64], except for the data of Zr_57_Nb_5_Cu_15.4_Ni_12.6_Al_10_, which were taken from [22,33]. Crossover temperature values, *T_A_*, were calculated using Equation (2). The glass transition temperatures of Hg, Ga, and Sn are taken from Table 1 of Reference [67]; the temperature of Na is taken from [68]; the temperature of K is taken from [69]; the temperature of Bi is taken from [70,71].

## Data Availability

The data supporting reported results can be provided upon request, except those contained in the references provided.
